# Ethinylestradiol in combined hormonal contraceptive has a broader effect on serum proteome compared with estradiol valerate: a randomized controlled trial

**DOI:** 10.1093/humrep/deac250

**Published:** 2022-11-23

**Authors:** M H Kangasniemi, R K Arffman, S Joenväärä, A Haverinen, K Luiro, T Tohmola, R Renkonen, O Heikinheimo, J S Tapanainen, T T Piltonen

**Affiliations:** Department of Obstetrics and Gynecology, Clinical Research Unit, Medical Research Center, Oulu University Hospital, University of Oulu, Oulu, Finland; Department of Obstetrics and Gynecology, Clinical Research Unit, Medical Research Center, Oulu University Hospital, University of Oulu, Oulu, Finland; Transplantation Laboratory, Haartman Institute, University of Helsinki, Helsinki, Finland; HUS Diagnostic Center, Helsinki University Hospital, Helsinki, Finland; Department of Obstetrics and Gynecology, University of Helsinki and Helsinki University Hospital, Helsinki, Finland; Department of Obstetrics and Gynecology, University of Helsinki and Helsinki University Hospital, Helsinki, Finland; Transplantation Laboratory, Haartman Institute, University of Helsinki, Helsinki, Finland; HUS Diagnostic Center, Helsinki University Hospital, Helsinki, Finland; Transplantation Laboratory, Haartman Institute, University of Helsinki, Helsinki, Finland; HUS Diagnostic Center, Helsinki University Hospital, Helsinki, Finland; Department of Obstetrics and Gynecology, University of Helsinki and Helsinki University Hospital, Helsinki, Finland; Department of Obstetrics and Gynecology, Clinical Research Unit, Medical Research Center, Oulu University Hospital, University of Oulu, Oulu, Finland; Department of Obstetrics and Gynecology, University of Helsinki and Helsinki University Hospital, Helsinki, Finland; Department of Obstetrics and Gynecology, Clinical Research Unit, Medical Research Center, Oulu University Hospital, University of Oulu, Oulu, Finland

**Keywords:** estradiol valerate, ethinylestradiol, combined contraceptive, proteome, randomized controlled trial, complement, acute phase signaling, metabolism, coagulation

## Abstract

**STUDY QUESTION:**

Does an estradiol-based combined oral contraceptive (COC) have a milder effect on the serum proteome than an ethinylestradiol (EE)-based COC or dienogest (DNG) only?

**SUMMARY ANSWER:**

The changes in serum proteome were multifold after the use of a synthetic EE-based COC compared to natural estrogen COC or progestin-only preparation.

**WHAT IS KNOWN ALREADY:**

EE-based COCs widely affect metabolism, inflammation, hepatic protein synthesis and blood coagulation. Studies comparing serum proteomes after the use of COCs containing EE and natural estrogens are lacking.

**STUDY DESIGN, SIZE, DURATION:**

This was a spin-off from a randomized, controlled, two-center clinical trial. Women (n = 59) were randomized to use either EE + DNG, estradiol valerate (EV) + DNG or DNG only continuously for 9 weeks.

**PARTICIPANTS/MATERIALS, SETTING, METHODS:**

Participants were healthy, young, white volunteer women. Serum samples were collected before and after 9 weeks of hormonal exposure. Samples from 44 women were available for analysis (EE + DNG n = 14, EV + DNG n = 16 and DNG only n = 14). Serum proteins were analyzed by quantitative, discovery-type label-free proteomics.

**MAIN RESULTS AND THE ROLE OF CHANCE:**

Altogether, 446 proteins/protein families with two or more unique peptides were detected and quantified. The number of proteins/families that altered over the 9-week period within the study groups was 121 for EE + DNG and 5 for EV + DNG, while no changes were detected for DNG only. When alterations were compared between the groups, significant differences were detected for 63 proteins/protein families, of which 58 were between the EE + DNG and EV + DNG groups. The most affected functions during the use of EE + DNG were the complement system, acute phase response signaling, metabolism and the coagulation system. The results were validated by fetuin-B and cortisol-binding globulin ELISA and sex hormone-binding globulin immunoassay.

**LARGE SCALE DATA:**

Data are available via ProteomeXchange with identifiers PXD033617 (low abundance fraction) and PXD033618 (high abundance fraction).

**LIMITATIONS, REASONS FOR CAUTION:**

The power analysis of the trial was not based on the proteomic analysis of this spin-off study. In the future, targeted proteomic analysis with samples from another trial should be carried out in order to confirm the results.

**WIDER IMPLICATIONS OF THE FINDINGS:**

The EE-based COC exerted a broader effect on the serum proteome than the EV-based COC or the DNG-only preparation. These results demonstrate that the effects of EE in COCs go far beyond the established endpoint markers of estrogen action, while the EV combination is closer to the progestin-only preparation. The study indicates that EV could provide a preferable option to EE in COCs in the future and signals a need for further studies comparing the clinical health outcomes of COCs containing EE and natural estrogens.

**STUDY FUNDING/COMPETING INTEREST(S):**

Funding for this researcher-initiated study was obtained from the Helsinki University Hospital research funds, the Hospital District of Helsinki and Uusimaa, the Sigrid Juselius Foundation, the Academy of Finland, the Finnish Medical Association, the University of Oulu Graduate School, the Emil Aaltonen Foundation, the Swedish Cultural Foundation in Finland, the Novo Nordisk Foundation, Orion Research Foundation and the Northern Ostrobothnia Regional Fund. The funders had no role in study design, data collection and analysis, publishing decisions or manuscript preparation. T.P. has received honoraria for lectures, consultations and research grants from Exeltis, Gedeon Richter, MSD, Merck, Pfizer, Roche, Stragen and Mithra Pharmaceuticals. O.H. occasionally serves on advisory boards for Bayer AG and Gedeon Richter and has designed and lectured at educational events for these companies. The other authors have nothing to disclose. O.H. occasionally serves on advisory boards for Bayer AG and Gedeon Richter and has designed and lectured at educational events for these companies. The other authors have nothing to disclose.

**TRIAL REGISTRATION NUMBER:**

ClinicalTrials.gov NCT02352090

**TRIAL REGISTRATION DATE:**

27 January 2015

**DATE OF FIRST PATIENT’S ENROLMENT:**

1 April 2015

## Introduction

In addition to the contraceptive effect, combined oral contraceptives (COCs) have vast effects on female physiology. In the liver, COCs stimulate the synthesis of steroid-binding globulins, such as sex hormone-binding globulin (SHBG), thereby affecting circulating, free steroid levels (Mashchak *et al.*, 1982; Wiegratz *et al.*, 2003; Sitruk-Ware *et al.*, 2007a,b; Piltonen *et al.*, 2012). COCs also increase low-grade inflammation, alter lipid metabolism and affect the coagulation system, resulting in an increased risk for thromboembolic events (van Rooijen *et al.*, 2006; Morin-Papunen *et al.*, 2008; Haarala *et al.*, 2009; Lidegaard *et al.*, 2009, 2012; Piltonen *et al.*, 2012; Wang *et al.*, 2016; Kangasniemi *et al.*, 2020). Even though millions of women worldwide use COCs, some of the effects and health outcomes, as well as their mode of action, remain poorly understood.

The net effect of COCs results from both estrogen and progestin action and their interplay (Sitruk-Ware and Nath, 2013). In COCs, ethinylestradiol (EE) is the most commonly used estrogen. It is a potent estrogen having up to 600 times greater effect on hepatic protein synthesis than natural estradiol (E2) (Mashchak *et al.*, 1982). EE provides efficient bleeding control in combination with different progestins; however, its oral dose is directly proportional to the risk of venous thrombosis (Sitruk-Ware and Nath, 2013; de Bastos *et al.*, 2014; Sitruk-Ware, 2016; Abou-Ismail *et al.*, 2020). Over recent decades, the dose of EE in COCs has been reduced to lower this risk.

Recently, natural estrogens have been included in COCs to reduce EE-related adverse events. New combinations containing E2, its ester estradiol valerate (EV), and estetrol (E4) have proven effective in combined contraceptives. They induce good cycle control and provoke less marked changes in metabolic and endocrine parameters compared with EE (Junge *et al.*, 2011; Sitruk-Ware and Nath, 2013; Stanczyk *et al.*, 2013; Mawet *et al.*, 2015; Grandi *et al.*, 2016; Kluft *et al.*, 2017; Haverinen *et al.*, 2021; Klipping *et al.*, 2021). However, most trials comparing COCs with different estrogens have included preparations that also differ in the progestin component; hence, knowledge of the specific estrogen effects in COCs is lacking. As the progestin component of COCs modulates the estrogen effects, the net effect of a COC depends on both the type of estrogen and the properties of the progestin (Sitruk-Ware and Nath, 2013; Abou-Ismail *et al.*, 2020).

Serum proteomic analysis provides an option to investigate the broad pharmacodynamic effects during medication use. Instead of specific, planned targets, this shotgun method analyzes all proteins and enables a broad overview and novel findings. To our knowledge, this is the first study to utilize such discovery-type proteomic analysis on hormonal contraceptives.

This study compared changes induced in the serum proteome during the use of COCs containing different estrogen components. A progestin-only preparation (containing the same progestin) was included as an active control. This study is a spin-off of a larger randomized controlled trial comparing the metabolic effects of EE + dienogest (DNG), EV + DNG and DNG only. We hypothesized that the EV + DNG preparation would have a milder impact on the serum proteome than that of EE + DNG and that the effect of DNG only would be neutral.

## Materials and methods

This randomized, controlled, investigator-initiated clinical trial was conducted at Helsinki and Oulu University Hospitals, Finland, between April 2015 and January 2018. The study protocol has been described previously (Haverinen *et al.*, 2021), and it was approved by the independent Ethics Committee of Helsinki University Central Hospital and the Finnish Medicines Agency. The regional ethics committee of the Northern Ostrobothnia Hospital District was informed of the approval. The study was registered with ClinicalTrials.gov (NCT02352090; https://clinicaltrials.gov/) and the EU Clinical Trials Register (EudraCT Number 2014-001243-20; https://www.clinicaltrialsregister.eu). All participants signed an informed consent form. The sample size calculation of the study was based on glucose metabolism, which was the trial’s primary outcome (Haverinen *et al.*, 2021).

### Subjects

**Figure 1.**
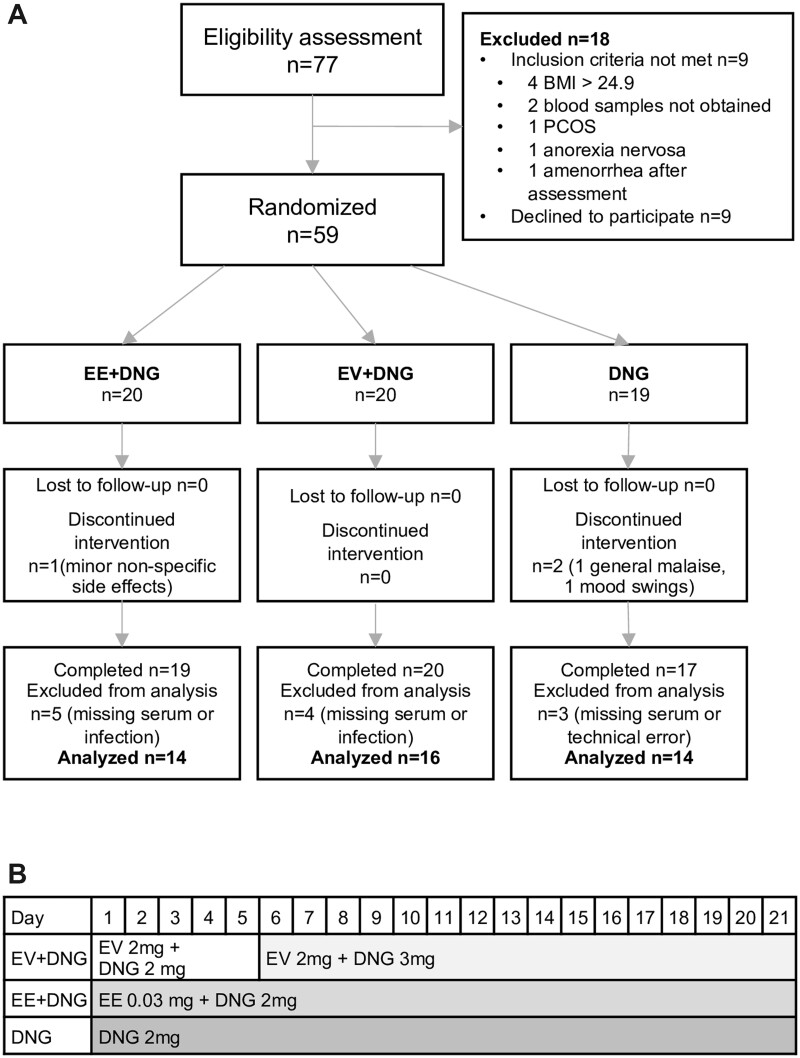
**Flow chart and study preparations.** Flow chart of the study (**A**) and hormone contents of the study preparations (**B**). The regimens were used continuously for 9 weeks. DNG, dienogest; EE, ethinylestradiol; EV, estradiol valerate.

Seventy-seven healthy women volunteered for the study (Fig. 1). After the eligibility assessment, 59 women were randomized. The participants had regular menstrual cycles and a minimum wash-out period of 2 months from hormonal medications and 3 months from breastfeeding. Exclusion criteria were age over 35 years, BMI ≥25 kg/m^2^, blood pressure ≥140/90 mmHg, smoking, alcohol or drug abuse, or any contraindication to COC use. Also, abnormalities in the 2-h oral glucose tolerance test or gynecological ultrasound examination led to exclusion from the study.

### Intervention

Participants were randomized to use either EE + DNG (Valette^®^, Bayer AG, Germany), EV + DNG (Qlaira^®^, Bayer AG, Germany) or DNG only (Visanne^®^, Jenapharm, Bayer AG, Germany) for 9 weeks continuously. Original packages were modified by removing placebo pills and pills containing only estrogen to minimize the differences in hormonal contents. The study flow chart and the exact hormonal contents of the study preparations are shown in Fig. 1. Serum samples were collected after a 12-h fast before the intervention on menstrual cycle days 1–5 and during the 9th week of treatment. After analysis for the primary and secondary outcomes of the original trial, serum samples for this spin-off study were available for 44 patients (EE + DNG n = 14, EV + DNG n = 16 and DNG n = 14).

### Serum proteomic analysis

Serum samples were stored at −70°C until analysis. High-abundance proteins and their protein complexes were affinity-purified from the samples, and the samples were divided into two pools: high- and low-abundance proteins. Human serum albumin was further removed from the high-abundance samples. Proteins were digested with trypsin and applied via a reverse-phase nano-liquid chromatograph column to a high-resolution mass spectrometer. Data were collected in a data-independent manner, and peptides were identified and quantified.

#### Affinity chromatography

An Agilent Technologies (Santa Clara, CA, USA) Multiple Affinity Removal Column Human 14 (Cat No 5188-6557) was used in the affinity chromatography, according to the manufacturer’s instructions. Samples were diluted four-fold with buffer A. Fourteen proteins and their complexes (i.e. ‘high-abundance proteins’) were retained in the column: albumin, IgG, IgA, transferrin, haptoglobin, antitrypsin, fibrinogen, alpha2-macroglobulin, alpha1-acid glycoprotein, IgM, apolipoprotein AI, apolipoprotein AII, complement C3 and transthyretin. Low-abundance proteins were collected as a flow-through fraction. High-abundance proteins were eluted with buffer B and collected.

#### Desalting and albumin removal from high-abundance samples

All samples were desalted using Thermo Fisher Scientific (Waltham, MA, USA) Zeba Spin desalting 96-well plates (Cat No 89808) to change the buffer to 25 mM Trizma BASE + 75 mM NaCl pH 7–8. The high-abundance samples were albumin-depleted with a Thermo Fisher Scientific Pierce Albumin Depletion Kit (Cat No 85160). Protein concentrations were measured in all samples using the Bradford method; 120 μg of proteins were aliquoted, dried, and stored at −70°C until digested. A SpeedVac vacuum concentrator (Model DNA120; Savant Systems LLC, MA, USA) was used for drying the samples.

#### Trypsin digestion

Proteins in the samples were trypsin-digested with the aid of Waters Corporation (Milford, MA, USA) 0.2% surfactant RapiGest™ SF. Samples were boiled for 10 min, and proteins were reduced by adding 0.5 M 1,4-dithiothreitol to a final concentration of 5 mM, vortexed and incubated for 30 min in 60°C water bath. In the next step, the proteins were alkylated with a 15 mM final concentration of iodoacetamide and incubated for 30 min in the dark at room temperature. Sequence grade trypsin Promega Biotech AB (Madison, WI, USA) Trypsin Gold (Cat No V5280) was added to the samples in a 1:100 enzyme-to-protein ratio. Samples were incubated at 37°C overnight. Samples were acidified to a final concentration of 0.5% trifluoroacetic acid and incubated for 45 min at 37°C, after which cleaved RapiGest™ SF was removed by a 10-min centrifugation at 16 000 *g* at 8°C. The supernatant was transferred to a new vial, and half of it was purified with Thermo Fisher Scientific Pierce C18 Spin Columns (Cat No 89870), according to the manufacturer’s instructions. The samples were dried and resuspended in 50 µl of 0.1% formic acid (FA) + 2% acetonitrile (ACN); 10 µl were used for peptide quantitation. Peptide concentrations were measured with a Thermo Fisher Scientific Pierce™ Quantitative Fluorometric Peptide Assay (Cat No 23290). The mass spectrometry samples were spiked in with a Waters Corporation Hi3 *E. Coli* Standard (Cat No 186006012), using 50 fmol/500 ng injected peptide mixture.

#### Mass spectrometry

Five hundred nanograms of the Hi3 spiked peptide mixture was injected to the Waters Corporation Synapt G2-Si HDMS and nanoACQUITY system. The system was equipped with Trap Column Symmetry C18, nanoACQUITY 10K 2G V/M Trap Column, 100 Å, 5 µm, 180 µm × 20 mm (Cat No 186006527) and analytical ultra-performance liquid chromatography (UPLC) column BEH C18 nanoACQUITY 10 K psi, 300 Å, 1.7 µm, 75 µm × 250 mm (Cat No 1886003815).

Samples were loaded to trapping column 8 µl/min for 2 minutes with 2% B buffer. The analytical gradient used was as follows: 0–1 min 2% B buffer, 1–65 min 55% B buffer, 65–80 min 80% B buffer, 80–87 min 80% B buffer, 87–90 min 2% B buffer and 90–100 min 2% B buffer. The gradient curve between the time points was linear. Analytical gradient flow rate was 200 nl/min. Buffer A was 0.1% FA in water, and buffer B was 0.1% FA in ACN. The gradient curve between the time points was linear. Buffer A was 0.1% FA in water, and buffer B was 0.1% FA in ACN. All solvents were mass spectrometry–grade from Merck Life Science (Darmstadt, Germany).

Data acquisition was performed with UPLC–ultra definition mass spectrometry (UPLC-UDMS^E^). Data were collected in the range of 100–2000 m/z, scan time 1 s, and ion mobility mass spectrometry wave velocity 650 m/s. Collision energy was ramped to fragment the peptides in the high-energy mode from 20 to 60 V. Calibration was performed with sodium iodide clusters over a mass range of 50–2500 m/z by infusing 2 µg/µl sodium iodide solution in 50/50 2-propanol/water into the mass spectrometer. At the same time, a leucine–enkephalin function for post-acquisition mass correction was collected (M+H^+^ 556.2771 m/z). In all, 10% of the samples were acquired as triplicates: the percentage coefficient of variation for the high-abundance proteins was 1.82, and for the low-abundance proteins 8.11.

#### Peptide identification and quantitation

The raw data were imported to Progenesis QI for proteomics (Nonlinear Dynamics, La Jolla, CA, USA), and simultaneously mass corrected with the leucine–enkephalin function. Proteinlynx Global Server (Waters Corporation) was used to identify peptides from the UDMS^E^-type data (Silva *et al.*, 2006a). In Progenesis, we used default parameters for peak picking and alignment, and the peptides were identified against Uniprot human FASTA sequences release 2019_06. A ClpB protein sequence (CLPB_ECOLI (P63285)) was inserted for label-free quantification to match the spike-in Hi3 standard. We used fixed modification at cysteine (carbamidomethyl) and variable at methionine (oxidation), and trypsin with two miss cleavages as digestion agents. The maximal false discovery rate was set to 1%, and fragment and peptide errors were set as automatic. The default parameters for ion matching were used: one or more ion fragments per peptide, three or more fragments per protein, and one or more peptides per protein. The proteins were assembled from the peptides by using the parsimony principle, and at least two unique peptides per protein were required. Progenesis used the method of Silva (Silva *et al.*, 2006b) for relative quantitation.

#### Hierarchical clustering and pathway analysis

Hierarchical clustering analysis was performed with MetaboAnalyst (https://www.metaboanalyst.ca/). QIAGEN Ingenuity Pathway Analysis (IPA) was used for pathway enrichment and network analysis. The desired protein accessions and fold changes were uploaded to the IPA, and canonical pathway enrichment was carried out with IPA Core Analysis. Default parameters were used for the analysis. The most significant pathway proteins were transferred to the network, and the proteins were connected where data were available in the Ingenuity Knowledge Base.

### Validation

For proteomic analysis validation, fetuin-B measurements were performed using ELISA. Serum samples were diluted 1:10 000 with DPBS (Corning #20-031-CVR, Mediatech, Inc., Manassas, VA, USA) and kit-specific dilution buffer, and analyzed with a FETUB ELISA Kit (Cat No EKX-LDKNLJ-96; Nordic BioSite AB, Täby, Sweden), according to the manufacturer’s instructions. Graphpad Prism 9 (GraphPad Software, San Diego, CA, USA) was used for interpolation of the data. Corticosteroid-binding globulin (CBG) was measured as part of the other sub-study (Kangasniemi *et al.*, 2022) using ELISA (Cat. No. RD192234200R, BioVendor, Brno, Czech Republic) and SHBG in another sub-study of the same trial (Haverinen *et al.*, 2022a) using a chemiluminescent microparticle immunoassay (Abbott Cat No 8K26, RRID: AB_2895255, Abbot Architect i2000SR analyzer, Abbot Diagnostics, IL, USA).

### Statistics

The distributions of protein changes were examined with the Shapiro–Wilk test. Most changes were skewed; thus, non-parametric tests were used for the analyses. The changes within the study groups were analyzed with the Wilcoxon test using the Benjamini–Hochberg procedure to control false discovery rates.

To investigate the differences between the study groups, we calculated the difference between the baseline and 9 weeks for all proteins. These differential variables were then analyzed with the Kruskal–Wallis test and the Benjamini–Hochberg procedure. Statistically significant results were further compared with the Mann–Whitney *post hoc* test, in which a value of *P* < 0.05 was considered significant. Wilcoxon test was performed using Python script from SciPY (Virtanen *et al.*, 2020) and other statistics using R package *stats* (R: A Language and Environment for Statistical Computing, R Foundation for Statistical Computing, Vienna, Austria).

## Results

### Study subjects

The baseline characteristics of the participants are shown in Table I. The randomization resulted in balanced study groups regarding age, BMI, blood pressure, glucose and lipid measurements.

**Table I:** Baseline characteristics of the study groups taking a 9-week course of combined oral contraceptive.

	EE + DNG	EV + DNG	DNG	Anova *P*-value*
	Mean ± SD	Mean ± SD	Mean ± SD	
Number of patients	14	16	14	
Age (years)	26 ± 4	24 ± 3	24 ± 3	0.17
BMI (kg/m^2^)	22.74 ± 1.78	22.53 ± 1.55	21.88 ± 2.07	0.42
WHR	0.79 ± 0.05	0.77 ± 0.04	0.78 ± 0.04	0.44
Systolic BP (mmHg)	118.93 ± 9.77	118.31 ± 7.59	111.57 ± 10.70	0.08
Diastolic BP (mmHg)	74.50 ± 8.26	74.06 ± 7.45	72.86 ± 7.85	0.85
Fasting glucose (mmol/l)	5.09 ± 0.35	5.21 ± 0.47	4.91 ± 0.38	0.14
HbA1c (mmol/mol)	32.79 ± 2.52	33.13 ± 2.66	32.14 ± 2.54	0.77
Fasting total cholesterol (mmol/l)	4.08 ± 0.60	3.93 ± 0.71	4.04 ± 0.47	0.19
HDL (mmol/l)	1.80 ± 0.41	1.59 ± 0.33	1.59 ± 0.26	0.58
LDL (mmol/l)	2.14 ± 0.51	2.17 ± 0.67	2.35 ± 0.49	0.95
Triglycerides (mmol/l)	0.67 ± 0.16	0.66 ± 0.25	0.64 ± 0.18	0.58
hs-CRP (mg/l)	0.98 ± 0.92	0.65 ± 0.54	0.56 ± 0.39	0.21

EE, ethinylestradiol; DNG, dienogest; EV, estradiol valerate; WHR, waist–hip ratio; BP, blood pressure; HDL, high-density lipoprotein; LDL, low-density lipoprotein; hs-CRP, high sensitivity C-reactive protein.

* Comparison between groups by one-way ANOVA.

### Proteomic analysis

#### Protein identification

A total of 446 proteins or protein families with two or more unique peptides were quantified. Some of the protein sequences are shared by many proteins (protein families), and some are unique in the human proteome. Depending on which peptides are identified, one can detect either a distinct protein or a protein family. Certain proteins were therefore identified and analyzed together as a family. Some proteins were detected both in high- and low-abundance analyses. The complete list of detected proteins is shown in Supplementary Table SI.

#### Hierarchical clustering

**Figure 2.**
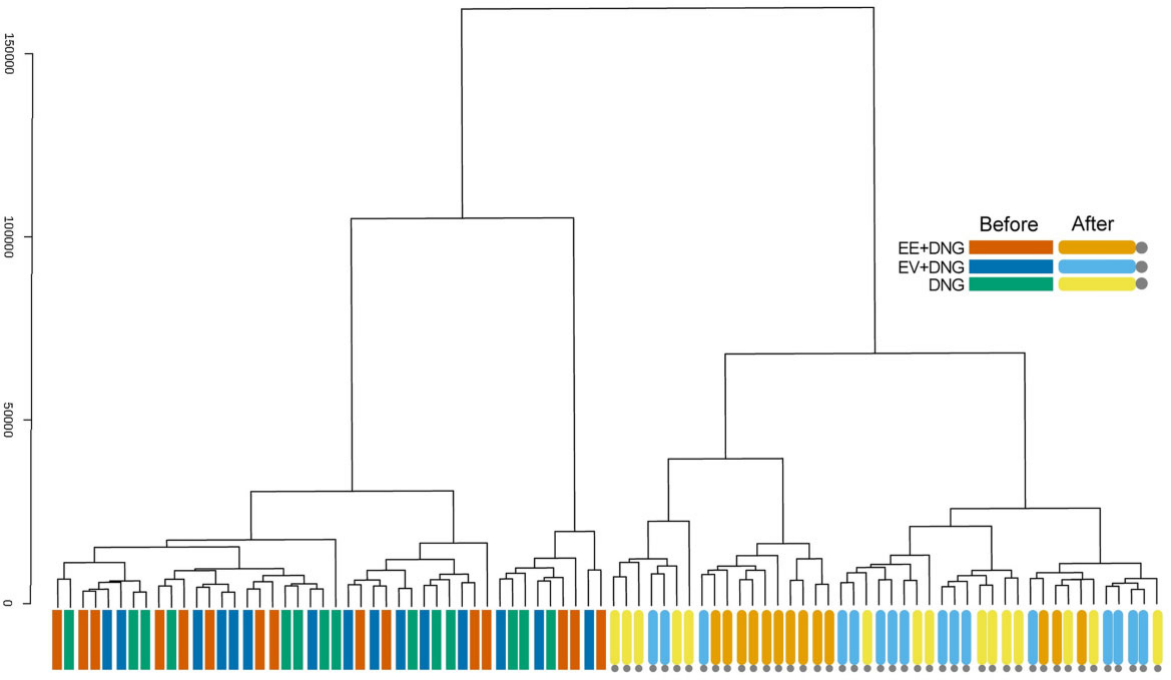
**Hierarchical clustering analysis based on the protein compositions of serum samples during a 9-week use of combined oral contraceptive.** Hierarchical clustering dendrogram of samples using the Euclidean distance measure (*Y*-axis) and the Ward clustering algorithm. The analysis shows that samples from the three study groups were mixed at baseline but after 9 weeks of use of the combined oral contraceptive preparations, the serum proteome profile clustered the participants according to preparation. The EE + DNG group in particular showed distinct clustering. The cluster was produced using MetaboAnalyst. DNG, dienogest; EE, ethinylestradiol; EV, estradiol valerate.

Successful randomization was further confirmed by hierarchical clustering based on protein compositions (Fig. 2). The 9-week intervention resulted in apparent clustering of the participants, especially the women using the EE + DNG preparation.

#### Proteome alterations within groups

**Figure 3.**
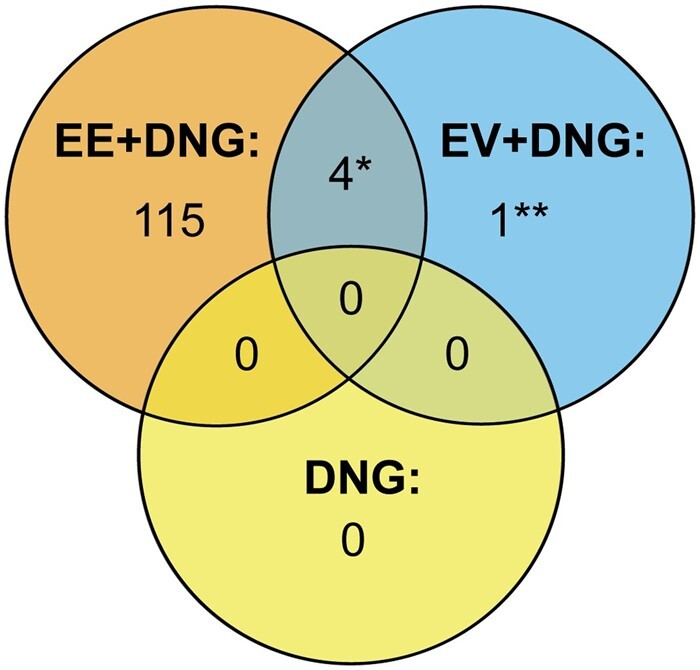
**Amounts of changing and unique proteins/protein families in serum from women taking a 9-week course of combined oral contraceptive.** Venn diagram showing the numbers of changed proteins after within-group analysis. DNG, dienogest; EE, ethinylestradiol; EV, estradiol valerate. *Interleukin1 receptor accessory protein, fetuin-B, angiotensinogen and ICOS ligand and Ig-like domain-containing protein. **Ectonucleotide pyrophosphatase/phosphodiesterase family member 2.

A total of 122 proteins/protein families changed significantly during the 9-week trial within at least one group: 121 in the EE + DNG and 5 in the EV + DNG group. No changes were observed in the DNG-only group. Some proteins were found in both low- and high-abundance fractions, indicating that proteins appeared both alone and in complexes with some high-abundance proteins, such as albumin. After consideration of these duplicate findings, the numbers of changing, unique proteins/families are shown in Fig. 3. All the proteins with relevant statistical data are compiled in Supplementary Table SI. The changes during EE + DNG use are further described in the ‘Pathway analysis’ section. Most of the changing proteins were related to inflammation, metabolism and coagulation. The five proteins that showed significant change during EV + DNG use were ectonucleotide pyrophosphatase/phosphodiesterase family member 2, ICOS ligand & Ig-like domain-containing protein, fetuin-B, angiotensinogen and interleukin-1 receptor accessory protein (IL1RAP).

#### Change in protein abundances between study groups

The change in protein abundance from baseline to 9 weeks was significantly different between the study groups for 63 proteins/protein families. In all, 58 proteins/protein families altered differently between the EE + DNG and EV + DNG groups, 61 between EE + DNG and DNG only and 17 between EV + DNG and DNG only. All significantly altered proteins between the groups are listed in Table II. Among the 10 most differently altered proteins, the highest fold changes were detected in CBG, SHBG, IL1RAP, angiotensinogen and fetuin-B.

**Table II:** Proteins with significantly differing alterations between study groups during the 9-week trial.

				Fold changes			Group comparison		
Fraction	Accession	Gene	Protein name	EE + DNG	EV + DNG	DNG	A	B	C
LOW	Q9NPH3	*IL1RAP*	Interleukin-1 receptor accessory protein	3.0*	1.7*	1.2	S	S	S
LOW	Q9UGM5	*FETUB*	Fetuin-B	2.0*	1.4*	1.2	S	S	S
LOW	P01019	*AGT*	Angiotensinogen	2.6*	1.7*	1.2	S	S	S
LOW	P04278	*SHBG*	Sex hormone-binding globulin	3.2*	1.4	1.2	S	S	NS
*LOW*	*O75144*	*ICOSLG*	*ICOS ligand*	2.2*	1.5*	*1.1*	*S*	*S*	*S*
	*A0A087X1L8*	*LOC102723996*	*Ig-like domain-containing protein*						
LOW	P08185	*SERPINA6*	Corticosteroid-binding globulin	3.2*	1.2	1.3	S	S	NS
LOW	Q9HD23	*MRS2*	Magnesium transporter MRS2 homolog, mitochondrial	2.1*	1.2	1.1	S	S	NS
LOW	P08294	*SOD3*	Extracellular superoxide dismutase [Cu-Zn]	0.5*	0.8	1.0	S	S	NS
LOW	Q96PD5	*PGLYRP2*	N-acetylmuramoyl-L-alanine amidase	0.5*	0.9	1.1	S	S	S
LOW	Q562R1	*ACTBL2*	Beta-actin-like protein 2	1.7*	1.2	1.1	S	S	NS
LOW	Q5TCS8	*AK9*	Adenylate kinase 9	0.5*	0.9	1.1	S	S	S
LOW	P04196	*HRG*	Histidine-rich glycoprotein	0.6*	0.9	1.1	S	S	NS
LOW	Q8N6C8	*LILRA3*	Leukocyte immunoglobulin-like receptor subfamily A member 3	0.6*	0.9	1.2	S	S	S
LOW	Q04756	*HGFAC*	Hepatocyte growth factor activator	0.5*	0.8	1.0	S	S	S
LOW	P01008	*SERPINC1*	Antithrombin-III	0.6*	0.9	1.1	S	S	S
LOW	P07225	*PROS1*	Vitamin K-dependent protein S	0.7*	1.0	1.1	S	S	NS
HIGH	P04083	*ANXA1*	Annexin A1	1.5*	1.1	1.0	S	S	NS
LOW	P05155	*SERPING1*	Plasma protease C1 inhibitor	1.1*	1.5	2.0	S	S	NS
HIGH	Q9NRF9	*POLE3*	DNA polymerase epsilon subunit 3	1.3*	1.0	1.0	S	S	NS
HIGH	Q9Y4X3	*CCL27*	C-C motif chemokine 27	1.3*	1.0	1.0	S	S	NS
LOW	P05452	*CLEC3B*	Tetranectin	0.6*	0.8	1.0	S	S	S
LOW	P07357	*C8A*	Complement component C8 alpha chain	0.8*	0.9	1.1	S	S	NS
LOW	P20851	*C4BPB*	C4b-binding protein beta chain	5.6*	6.1	7.0	S	S	NS
HIGH	A4FU69	*EFCAB5*	EF-hand calcium-binding domain-containing protein 5	0.9*	1.0	1.1	S	S	NS
HIGH	P20742	*PZP*	Pregnancy zone protein	1.5*	1.0	1.0	S	S	NS
*LOW*	*P06396*	*GSN*	*Gelsolin*	0.7*	*0.9*	*1.1*	*S*	*S*	*NS*
	*P22460*	*KCNA5*	*Voltage-gated potassium channel HK2*						
	*Q8N1V2*	*CFAP52*	*Cilia- and flagella-associated protein 52*						
	*Q99607*	*ELF4*	*ETS-related transcription factor Elf-4*						
	*Q9UH62*	*ARMCX3*	*Armadillo repeat-containing X-linked protein 3*						
	*Q9Y2M5*	*KLHL20*	*Kelch-like protein 20*						
HIGH	P01009	*SERPINA1*	Alpha-1-antitrypsin	1.4*	1.0	1.0	S	S	NS
LOW	A5A3E0	*POTEF*	POTE ankyrin domain family member F	0.6*	0.8	1.0	S	S	S
LOW	P02747	*C1QC*	Complement C1q subcomponent subunit C	0.8*	0.9	1.1	NS	S	S
LOW	Q92835	*INPP5D*	Phosphatidylinositol 3,4,5-trisphosphate 5-phosphatase 1	0.8*	0.9	1.1	S	S	NS
HIGH	P01880	*IGHD*	Immunoglobulin heavy constant delta	0.8*	1.0	1.0	S	S	NS
LOW	P04003	*C4BPA*	C4b-binding protein alpha chain	1.9*	2.1	2.8	S	S	NS
LOW	P51884	*LUM*	Lumican	0.6*	0.8	1.0	S	S	NS
LOW	P02749	*APOH*	Beta-2-glycoprotein 1	331.5*	443.3	589.0	S	S	NS
LOW	P07737	*PFN1*	Profilin-1	0.7*	0.9	1.1	S	S	NS
LOW	P09871	*C1S*	Complement C1s subcomponent	0.8*	0.9	1.1	S	S	NS
LOW	P13671	*C6*	Complement component C6	0.8*	0.9	1.0	S	S	NS
LOW	P02748	*C9*	Complement component C9	0.7*	0.9	1.0	S	S	NS
LOW	P08697	*SERPINF2*	Alpha-2-antiplasmin	0.8*	1.0	1.1	S	S	NS
*LOW*	*P01011*	*SERPINA3*	*Alpha-1-antichymotrypsin*	0.7*	*0.9*	*1.1*	*NS*	*S*	*NS*
	*Q9HCY8*	*S100A14*	*Protein S100-A14*						
LOW	P06727	*APOA4*	Apolipoprotein A-IV	10.7*	13.0	17.2	NS	S	S
LOW	Q16473	*TNXA*	Putative tenascin-XA	0.6*	0.8	1.0	S	S	NS
LOW	Q13822	*ENPP2*	Ectonucleotide pyrophosphatase/phosphodiesterase family member 2	1.5	0.8*	1.0	S	NS	S
LOW	P02745	*C1QA*	Complement C1q subcomponent subunit A	0.7*	0.9	1.1	S	S	NS
*LOW*	*P00450*	*CP*	*Ceruloplasmin*	1.5*	*1.1*	*1.2*	*S*	*S*	*NS*
	*Q96L14*	*CEP170P1*	*Cep170-like protein*						
LOW	Q9BWP8	*COLEC11*	Collectin-11	0.7*	1.0	1.1	S	S	NS
LOW	P35442	*THBS2*	Thrombospondin-2	0.7*	0.9	1.0	S	S	NS
LOW	Q9NQ79	*CRTAC1*	Cartilage acidic protein 1	0.7*	0.9	1.0	S	S	NS
LOW	P00736	*C1R*	Complement C1r subcomponent	0.8*	0.9	1.1	S	S	NS
LOW	P02746	*C1QB*	Complement C1q subcomponent subunit B	0.7*	0.9	1.1	S	S	NS
LOW	P07360	*C8G*	Complement component C8 gamma chain	0.8*	1.0	1.1	S	S	NS
LOW	P02776	*PF4*	Platelet factor 4	1.1	0.9	1.1	S	NS	S
HIGH	P08185	*SERPINA6*	Corticosteroid-binding globulin	1.4*	1.0	1.0	S	S	NS
LOW	P09172	*DBH*	Dopamine beta-hydroxylase	0.7*	0.9	1.1	S	S	NS
LOW	P10643	*C7*	Complement component C7	0.8*	0.9	1.1	S	S	NS
LOW	Q8WXW3	*PIBF1*	Progesterone-induced-blocking factor 1	0.7*	1.0	1.0	S	S	NS
LOW	Q8N531	*FBXL6*	F-box/LRR-repeat protein 6	0.8*	1.0	1.0	S	S	NS
LOW	P05019	*IGF1*	Insulin-like growth factor I	0.8*	0.9	1.1	S	S	NS
LOW	P05543	*SERPINA7*	Thyroxine-binding globulin	1.4*	1.1	1.1	S	S	NS
LOW	Q6EMK4	*VASN*	Vasorin	0.7*	0.9	1.1	S	S	NS
LOW	Q9UBP9	*GULP1*	PTB domain-containing engulfment adapter protein 1	0.8	0.9	1.1	NS	S	S
LOW	Q96QU8	*XPO6*	Exportin-6	0.7*	0.9	1.1	S	S	NS
HIGH	Q9H078	*CLPB*	Caseinolytic peptidase B protein homolog	0.6*	0.9	1.0	NS	S	NS

Proteins with a statistically different change between the groups after the Kruskal–Wallis test and Benjamini–Hochberg method (in order of significance). The Mann–Whitney *U* test was used for pairwise analysis. Proteins shown in italics belong to the family of proteins with similar peptides with shared statistical analysis. The high-abundance fraction indicates that the protein co-eluted with high-abundance proteins, i.e. it had probably formed a complex with highly concentrated proteins in the serum. A fold change >1 indicates an increase in protein amount, whereas a fold change between 0 and 1 reflects reduced quantity.

* Statistically significant change within the group.

A: ethinylestradiol (EE) + dienogest (DNG) versus estradiol valerate (EV) + DNG. B: EE + DNG versus DNG. C: EV + DNG versus DNG. S: Mann–Whitney *U* test *P* < 0.05; NS, non-significant.

#### Pathway analysis

**Figure 4.**
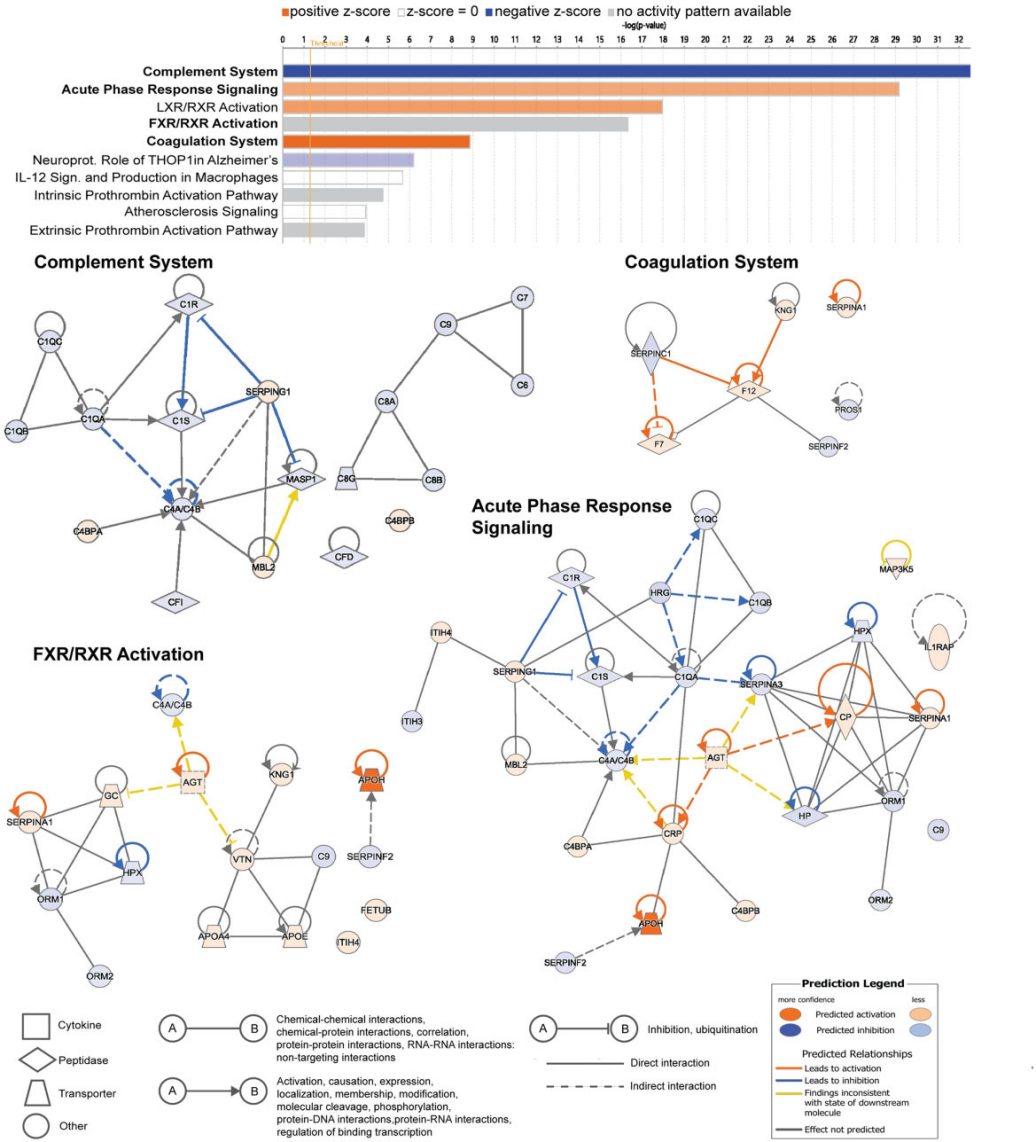
**Pathway and network analyses of changing proteins during EE + DNG use.** Pathway analysis of the 10 pathways most affected during 9-week use of ethinylestradiol + dienogest (EE + DNG). Graph shows category scores; ‘threshold’ indicates the minimum significance level (scored as −log(*P*-value) from Fisher’s exact test, set here to 1.25). A positive z-score (orange bar) predicts activation of the pathway, whereas a negative (blue bar) predicts inhibition. The analysis was performed with QIAGEN Ingenuity Pathway Analysis (IPA) software. The four pathways in bold text are depicted below as networks. FXR, farnesoid X receptor; LXR, liver X receptor; RXR, retinoid X receptor.

Most changes in the serum proteins were detected in the EE + DNG group; therefore, the functionality analysis was based on the proteins with a significant change during this treatment. The 10 most significantly affected pathways are listed in Fig. 4. The use of EE + DNG led to predicted inhibition of the complement system and activation of acute phase response signaling, liver X receptor/retinoid X receptor (LXR/RXR) and the coagulation system. The detected proteins of the farnesoid X receptor (FXR)/RXR activation pathway were 88% the same as in the LXR/RXR activation pathway. The complement system was the most-affected pathway, with 19 significantly changed proteins. The proteins belonging to the five pathways most changed during EE + DNG treatment are shown in Supplementary Table SII.

Full lists of normalized abundance data are included in Supplementary Tables SIII (high-abundance protein fraction) and SIV (low-abundance protein fraction).

### Fetuin-B, CBG and SHBG

**Figure 5.**
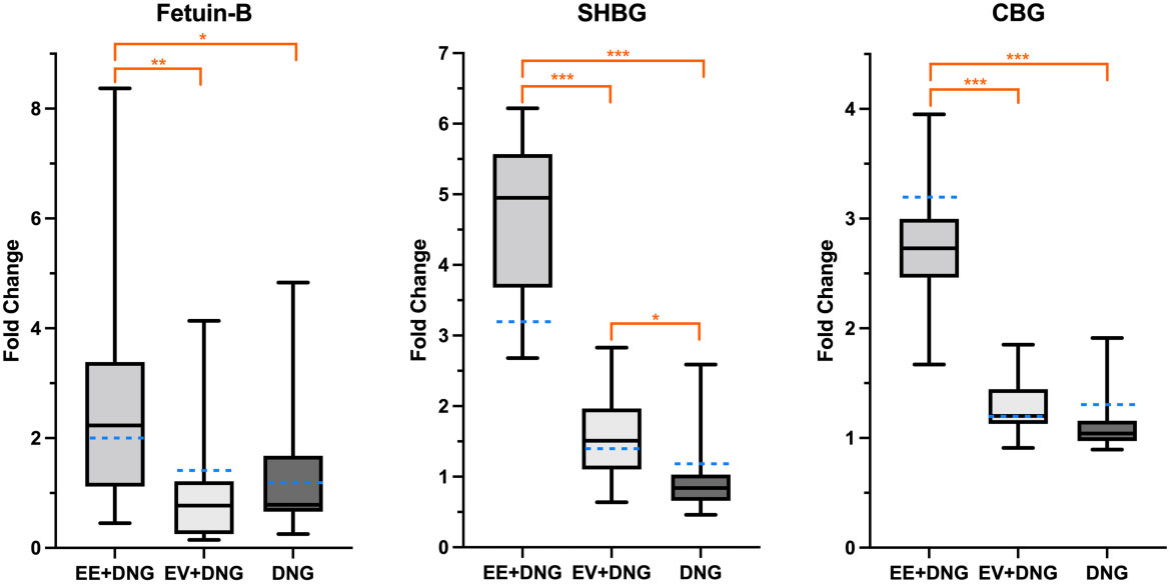
**Validation of proteomic analysis of serum from women taking a 9-week course of combined oral contraceptive.** The fold changes in serum Fetuin-B, CBG and SHBG during the trial, as measured by ELISA and immunoassay. Box blots present the fold changes gained from the validation analyses, whereas the blue dotted lines show the fold changes from the proteomic analysis. The fetuin-B concentration increased especially during use of the combined oral contraceptive containing ethinylestradiol + dienogest (EE + DNG). One major fetuin-B outlier was removed from the EE + DNG group, which did not affect the statistical analysis. CBG and SHBG concentrations have been reported previously (Kangasniemi *et al.*, 2022; Haverinen *et al.*, 2022a). DNG, dienogest; EE, ethinylestradiol; EV, estradiol valerate; CBG, cortisol-binding globulin; SHBG, sex hormone-binding globulin. *P < 0.05; **P < 0.01; ***P < 0.001.

After proteomic analysis, fetuin-B (a novel finding with a significant difference between the study groups) was chosen for additional validation. We previously reported the changes in CBG (Kangasniemi *et al.*, 2022) and SHBG (Haverinen *et al.*, 2022a) and those analyses were also used for validation of proteomic analysis (Fig. 5). For fetuin-B, the median change (95% CI, *P*-value for change within the group) during the trial was the following: EE + DNG 206.04 ng/ml ((92.86–410.6) *P *=* *0.02), EV + DNG −52.20 ng/ml ((−77.92–22.83) *P *=* *0.112) and DNG −12.62 ng/ml ((−81.84–185.44) *P *=* *0.73). In the proteomics analysis, EE + DNG induced a higher increase in serum CBG and SHBG compared with the other study preparations. These findings are in line with our previous ELISA and immunoassay results (Kangasniemi *et al.*, 2022; Haverinen *et al.*, 2022a); the fold changes derived from these data are shown in Fig. 5.

## Discussion

This study showed that 9 weeks of EE-based COC use had a multifold effect on the serum proteome compared with EV-based COC or DNG-only preparation; the number of affected proteins after exposure to EE + DNG was 24-fold the number for EV + DNG and 120-fold that for DNG only. Few proteins changed during the use of EV + DNG and no changes were detected during the use of DNG only. The most-affected pathways were the complement system, acute phase response signaling, metabolism-related LXR&FXR/RXR activation and the coagulation system. The findings of the proteomic analysis were validated with fetuin-B ELISA and our previously reported CBG ELISA and SHBG immunoassay, showing that treatment with EE + DNG increased serum levels of fetuin-B, CBG and SHBG more than EV + DNG or DNG only.

To our knowledge, this is the first study to explore the effects of hormonal contraceptives on serum proteins with an untargeted, discovery-type proteomic analysis. Previously, only one study has utilized a proteomic analysis method during contraceptive use (Josse *et al.*, 2012). However, that cross-sectional study had selected targets for analysis and compared hormonal contraceptive use to non-use but did not specify the treatments. Similar to our data, differences were observed in proteins related to inflammation, innate immunity, coagulation, and blood pressure regulation (Josse *et al.*, 2012). Quantitation in the discovery-type analysis is not as accurate as in the targeted method, but it offers a wider, indicative insight into the matter in question. The next validating step for the discovery-type analysis would be targeted analysis based on the results of this study with a new sample set from other subjects. However, many of the effects seen in this study have been previously observed with other targeted methods, which supports the reliability of the results.

EE-containing COCs have been shown to increase low-grade inflammation, reflected by increased levels of C-reactive protein (CRP) (van Rooijen *et al.*, 2006; Morin-Papunen *et al.*, 2008; Haarala *et al.*, 2009; Piltonen *et al.*, 2012; Wang *et al.*, 2016), which is a well-known marker of the acute phase. Activation of the acute phase response signaling pathway by EE + DNG was also detected in the present study (Fig. 4), exemplified by the increase in IL1RAP and angiotensinogen, a precursor of the vasoconstrictor angiotensin. EV + DNG also increased these proteins, but the increase was significantly milder compared with EE + DNG. Similarly, in our earlier study, EE + DNG had a greater effect on the inflammatory markers CRP and pentraxin 3 compared with EV + DNG (Kangasniemi *et al.*, 2020). These inflammatory changes are relevant as regards the cardiovascular morbidity related to COC use compared to progestin-only preparations, as shown in a recent review (Okoth *et al.*, 2020). To support this, moderate effects of natural estrogens were also shown in a study comparing EE + drospirenone (DRSP) and E4 + DRSP, which found a greater increase in angiotensinogen during the use of EE + DRSP (Klipping *et al.*, 2021). In the same study, EE + DRSP also increased CRP, although the difference compared to E4 + DRSP did not reach statistical significance (Klipping *et al.*, 2021).

EE is a highly potent estrogen known to have a major effect on hepatic protein synthesis: EE increases SHBG, CBG and angiotensinogen multifold compared with estradiol (Mashchak *et al.*, 1982). Previous studies have demonstrated increases in angiotensinogen and binding globulins during the use of EE-based combined contraceptives, regardless of the administration route (Sitruk-Ware *et al.*, 2007b; Piltonen *et al.*, 2012; Westhoff *et al.*, 2013; Klipping *et al.*, 2021). However, the impact of COCs containing natural estrogens on hepatic protein synthesis appears to be lower than that of EE-based COCs. This was shown in a comparative study in which E4 + DRSP promoted SHBG and CBG synthesis less than EE + DRSP (Klipping *et al.*, 2021). The lower impact of natural estrogens on hormone-binding globulins has also been observed comparing EV + DNG with EE + levonorgestrel (LNG) (Junge *et al.*, 2011), and E2 + nomegestrol acetate with EE + LNG (Ågren *et al.*, 2011). These studies indicate a lesser hepatic impact of natural estrogens, supporting the results of the present study. It is also well known that COCs increase the risk of thromboembolic events (Sitruk-Ware *et al.*, 2007a; Lidegaard *et al.*, 2009, 2012; de Bastos *et al.*, 2014). Interestingly, new data suggest that the effects on coagulation and the risk for thromboembolism might be lower with natural estrogen combinations (Sitruk-Ware, 2016; Kluft *et al.*, 2017; Heikinheimo *et al.*, 2022; Haverinen *et al.*, 2022b), which may be related to the lesser impact on the liver.

In the pathway analysis, as many as 19 proteins related to the complement system were altered during EE + DNG treatment, which predicts inhibition of this pathway. The complement system is a complex immunological system involving ∼30 interacting proteins that compose a cascade of activation steps leading to activation of inflammation, opsonization of pathogens and cells for destruction, and direct killing of target cells or microbes by lysis (Oksjoki *et al.*, 2007). The complement system has a central role in the immune system by discriminating healthy tissues from apoptotic cells and microbes, but its imbalance and uncontrolled activation is associated with various diseases, such as glomerulonephritis, sepsis and cardiovascular diseases (Oksjoki *et al.*, 2007; Ricklin *et al.*, 2010). Complement components C3 and C4 in serum increase during pregnancy (He *et al.*, 2020), which is a high estrogen state. During pregnancy, complement dysregulation may lead to miscarriage, pre-eclampsia or pre-term birth (Girardi *et al.*, 2020). As shown above, COCs are known to alter the inflammatory milieu, but data are limited on their effects on the complement system. A study from 1975 showed increases in C3 and decreases in CH50, C6, and C7 during estrogen–progestogen contraceptive use compared with women not using hormonal contraceptives (Tedder *et al.*, 1975). Estrogen-regulated complement action has recently been demonstrated by showing that estrogen promotes an adaptation response in *Candida albicans*: through inhibition of complement-mediated opsonophagocytosis, *C. albicans* can evade the innate immune system (Kumwenda *et al.*, 2022). In rheumatoid arthritis, lower circulating levels of properdin, one complement component, were measured in women who had ever used COCs compared with never-users (Bemis *et al.*, 2019). Inflammatory activation of EE is unknown, but the effects on the complement cascade probably contribute to this process and should be further studied.

Untargeted proteomic analysis also enables the identification of interesting novel targets for future research. Fetuin-B is a relatively understudied hepatokine associated with insulin resistance, dyslipidemia, and non-alcoholic fatty liver disease (Meex and Watt, 2017; Mokou *et al.*, 2020; Olkowicz *et al.*, 2021). Serum levels of fetuin-B increase during pregnancy but normalize after delivery (Šimják *et al.*, 2018), possibly related to hormonal changes of pregnancy. One study investigated fetuin-B levels in four women taking COCs containing EE in combination with LNG, DRSP, or DNG, all of which increased fetuin-B levels (Floehr *et al.*, 2016). In a cross-over model, one woman used DNG only first and continued with EE + DNG: fetuin-B levels remained unchanged during DNG only but increased after initiation of EE + DNG treatment (Floehr *et al.*, 2016). These data are in line with our results. Fetuin-B is part of the FXR/RXR activation pathway (Fig. 4), which is closely related to the LXR/RXR activation pathway (Redinger, 2003). Both of these pathways, also affected by EE + DNG in the present study, play critical roles in regulating lipid and glucose metabolism (Edwards *et al.*, 2002; Redinger, 2003; Wang *et al.*, 2008). EE-containing contraceptives alter lipid metabolism, increasing especially high-density lipoprotein and triglycerides, but also affect glucose metabolism (Morin-Papunen *et al.*, 2008; Piltonen *et al.*, 2012; Wang *et al.*, 2016). Whether the activations of the FXR/RXR and LXR/RXR pathways are mediators of these changes remains to be studied. If this is confirmed, natural estrogens might also be less detrimental in this aspect (Junge *et al.*, 2011; Mawet *et al.*, 2015; Kangasniemi *et al.*, 2020; Klipping *et al.*, 2021).

The main strength of the present study is that this randomized trial is the first comparison of EE- and EV-containing contraceptives combined with the same progestin. Randomization was successful regarding the clinical characteristics but also at the proteome level, as reflected in the mixed clustering of baseline samples. Therefore, the changes observed during the trial are likely related to the intervention and not to chance. However, we acknowledge that the 9-week study period is relatively short for concluding long-term effects. Therefore, these findings should be further examined in longer trials with specifically targeted measurements. Proteomic analysis was not considered in the original sample size calculation, given that the analysis was not the primary outcome. On the other hand, as no previous data exist, estimating power for a discovery-type setup is extremely challenging. However, in the field of discovery-type proteomics, the groups of 14 are quite extensive, especially considering the trial with repeated measurements. Despite these limitations, our results give valuable insight into the extent of the differences between the estrogen types in COCs.

To conclude, EE + DNG had a greater impact on the serum proteome than that of EV + DNG or the DNG-only preparation. The differences between EE and EV go far beyond the well-known, traditional estrogen actions. Therefore, EV could provide a preferable option to EE in COCs in the future. Moreover, although our findings need to be validated in further studies utilizing targeted methods, this study provides a valuable basis for future research and guides the development of hormonal contraceptives.

## Supplementary Material

deac250_Supplementary_Table_SI

deac250_Supplementary_Table_SII

deac250_Supplementary_Table_SIII

deac250_Supplementary_Table_SIV

## Data Availability

Normalized abundance data of high- and low-abundance fractions are included in Supplementary Tables SIII and SIV. The mass spectrometry proteomics data have been deposited to the ProteomeXchange Consortium via the PRIDE (Perez-Riverol *et al.*, 2022) partner repository with the dataset identifiers PXD033617 and 10.6019/PXD033617 for the low-abundance proteins and PXD033618 and 10.6019/PXD033618 for the high fraction.

## References

[deac250-B1] Abou-Ismail MY , Citla Sridhar D, Nayak L. Estrogen and thrombosis: a bench to bedside review. Thromb Res 2020;192:40–51. 32450447 10.1016/j.thromres.2020.05.008 PMC7341440

[deac250-B2] Ågren UM , Anttila M, Mäenpää-Liukko K, Rantala ML, Rautiainen H, Sommer WF, Mommers E. Effects of a monophasic combined oral contraceptive containing nomegestrol acetate and 17β-oestradiol in comparison to one containing levonorgestrel and ethinylestradiol on markers of endocrine function. Eur J Contracept Reprod Health Care 2011;16:458–467. 21942708 10.3109/13625187.2011.614363 PMC3233273

[deac250-B3] Bemis EA , Norris JM, Seifert J, Frazer-Abel A, Okamoto Y, Feser ML, Demoruelle MK, Deane KD, Banda NK, Holers VM. Complement and its environmental determinants in the progression of human rheumatoid arthritis. Mol Immunol 2019;112:256–265. 31207549 10.1016/j.molimm.2019.05.012 PMC7712508

[deac250-B4] de Bastos M , Stegeman BH, Rosendaal FR, van HVA, Helmerhorst FM, Stijnen T, Dekkers OM. Combined oral contraceptives: venous thrombosis. Cochrane Database Syst Rev 2014;2014:CD010813. 10.1002/14651858.CD010813.pub2 PMC10637279 24590565

[deac250-B5] Edwards PA , Kennedy MA, Mak PA. LXRs; Oxysterol-activated nuclear receptors that regulate genes controlling lipid homeostasis. Vascul Pharmacol 2002;38:249–256. 12449021 10.1016/s1537-1891(02)00175-1

[deac250-B6] Floehr J , Dietzel E, Neulen J, Rösing B, Weissenborn U, Jahnen-Dechent W. Association of high fetuin-B concentrations in serum with fertilization rate in IVF: a cross-sectional pilot study. Hum Reprod 2016;31:630–637. 26759143 10.1093/humrep/dev340

[deac250-B7] Girardi G , Lingo JJ, Fleming SD, Regal JF. Essential role of complement in pregnancy: from implantation to parturition and beyond. Front Immunol 2020;11:1681. 32849586 10.3389/fimmu.2020.01681 PMC7411130

[deac250-B8] Grandi G , Napolitano A, Cagnacci A. Metabolic impact of combined hormonal contraceptives containing estradiol. Expert Opin Drug Metab Toxicol 2016;12:779–787. 27187588 10.1080/17425255.2016.1190832

[deac250-B9] Haarala A , Eklund C, Pessi T, Lehtimäki T, Huupponen R, Jula A, Viikari J, Raitakari O, Hurme M. Use of combined oral contraceptives alters metabolic determinants and genetic regulation of C‐reactive protein. The Cardiovascular Risk in Young Finns Study. Scand J Clin Lab Invest 2009;69:168–174. 18937150 10.1080/00365510802449642

[deac250-B10] Haverinen A , Kangasniemi MH, Luiro K, Piltonen T, Heikinheimo O, Tapanainen JS. Ethinyl estradiol vs estradiol valerate in combined oral contraceptives—effect on glucose tolerance: a randomized, controlled clinical trial. Contraception 2021;103:53–59. 33098852 10.1016/j.contraception.2020.10.014

[deac250-B11] Haverinen A , Luiro K, Kangasniemi MH, Piltonen TT, Hustad S, Heikinheimo O, Tapanainen JS. Estradiol valerate vs ethinylestradiol in combined oral contraceptives: effects on the pituitary-ovarian axis. J Clin Endocrinol Metab2022a;107:e3008–e3017. 35279718 10.1210/clinem/dgac150

[deac250-B12] Haverinen A , Luiro KM, Szanto T, Kangasniemi MH, Hiltunen L, Sainio S, Piltonen TT, Lassila R, Tapanainen JS, Heikinheimo O. Combined oral contraceptives containing estradiol valerate vs ethinylestradiol on coagulation: a randomized clinical trial. Acta Obstet Gynecol Scand2022b;101:1102–1111. 35909329 10.1111/aogs.14428 PMC9812067

[deac250-B13] He YD , Xu BN, Song D, Wang YQ, Yu F, Chen Q, Zhao MH. Normal range of complement components during pregnancy: a prospective study. Am J Reprod Immunol 2020;83:e13202. 31646704 10.1111/aji.13202 PMC7027513

[deac250-B14] Heikinheimo O , Toffol E, Partonen T, But A, Latvala A, Haukka J. Systemic hormonal contraception and risk of venous thromboembolism. Acta Obstet Gynecol Scand 2022;101:846–855. 35633036 10.1111/aogs.14384 PMC9564731

[deac250-B15] Josse AR , Garcia-Bailo B, Fischer K, El-Sohemy A. Novel effects of hormonal contraceptive use on the plasma proteome. PLoS One 2012;7:e45162. 22984625 10.1371/journal.pone.0045162 PMC3440362

[deac250-B16] Junge W , Mellinger U, Parke S, Serrani M. Metabolic and haemostatic effects of estradiol valerate/dienogest, a novel oral contraceptive: a randomized, open-label, single-centre study. Clin Drug Investig 2011;31:573–584. 10.2165/11590220-000000000-00000 21721593

[deac250-B17] Kangasniemi MH , Arffman RK, Haverinen A, Luiro K, Hustad S, Heikinheimo O, Tapanainen JS, Piltonen TT. Effects of estradiol- and ethinylestradiol-based contraceptives on adrenal steroids: a randomized trial. Contraception 2022;https://doi.org/10.1016/j.contraception.2022.08.009. 10.1016/j.contraception.2022.08.009 36084710

[deac250-B18] Kangasniemi MH , Haverinen A, Luiro K, Hiltunen JK, Komsi EK, Arffman RK, Heikinheimo O, Tapanainen JS, Piltonen TT. Estradiol valerate in COC has more favorable inflammatory profile than synthetic ethinyl estradiol: a randomized trial. J Clin Endocrinol Metab 2020;105:e2483–e2490. 10.1210/clinem/dgaa186 32303765

[deac250-B19] Klipping C , Duijkers I, Mawet M, Maillard C, Bastidas A, Jost M, Foidart JM. Endocrine and metabolic effects of an oral contraceptive containing estetrol and drospirenone. Contraception 2021;103:213–221. 33428907 10.1016/j.contraception.2021.01.001

[deac250-B20] Kluft C , Zimmerman Y, Mawet M, Klipping C, Duijkers IJM, Neuteboom J, Foidart JM, Bennink HC. Reduced hemostatic effects with drospirenone-based oral contraceptives containing estetrol vs. ethinyl estradiol. Contraception 2017;95:140–147. 27593335 10.1016/j.contraception.2016.08.018

[deac250-B21] Kumwenda P , Cottier F, Hendry AC, Kneafsey D, Keevan B, Gallagher H, Tsai HJ, Hall RA. Estrogen promotes innate immune evasion of *Candida albicans* through inactivation of the alternative complement system. Cell Rep 2022;38:110183. 34986357 10.1016/j.celrep.2021.110183 PMC8755443

[deac250-B22] Lidegaard Ø , Lokkegaard E, Jensen A, Skovlund CW, Keiding N. Thrombotic stroke and myocardial infarction with hormonal contraception. N Engl J Med 2012;366:2257–2266. 22693997 10.1056/NEJMoa1111840

[deac250-B23] Lidegaard Ø , Løkkegaard E, Svendsen AL, Agger C. Hormonal contraception and risk of venous thromboembolism: national follow-up study. BMJ 2009;339:b2890. 19679613 10.1136/bmj.b2890 PMC2726928

[deac250-B24] Mashchak CA , Lobo RA, Dozono-Takano R, Eggena P, Nakamura RM, Brenner PF, Mishell DR. Comparison of pharmacodynamic properties of various estrogen formulations. Am J Obstet Gynecol 1982;144:511–518. 6291391 10.1016/0002-9378(82)90218-6

[deac250-B25] Mawet M , Maillard C, Klipping C, Zimmerman Y, Foidart JM, Bennink HJTC. Unique effects on hepatic function, lipid metabolism, bone and growth endocrine parameters of estetrol in combined oral contraceptives. Eur J Contracept Reprod Health Care 2015;20:463–475. 26212489 10.3109/13625187.2015.1068934 PMC4699469

[deac250-B26] Meex RCR , Watt MJ. Hepatokines: linking nonalcoholic fatty liver disease and insulin resistance. Nat Rev Endocrinol 2017;13:509–520. 28621339 10.1038/nrendo.2017.56

[deac250-B27] Mokou M , Yang S, Zhan B, Geng S, Li K, Yang M, Yang G, Deng W, Liu H, Liu D et al Elevated circulating fetuin-B levels are associated with insulin resistance and reduced by GLP-1RA in newly diagnosed PCOS women. Mediators Inflamm 2020;2020:2483435. 33061822 10.1155/2020/2483435 PMC7545451

[deac250-B28] Morin-Papunen L , Martikainen H, McCarthy MI, Franks S, Sovio U, Hartikainen A-L, Ruokonen A, Leinonen M, Laitinen J, Järvelin M-R et al Comparison of metabolic and inflammatory outcomes in women who used oral contraceptives and the levonorgestrel-releasing intrauterine device in a general population. Am J Obstet Gynecol 2008;199:529.e1–529.e10. 10.1016/j.ajog.2008.04.013 18533124

[deac250-B29] Okoth K , Chandan JS, Marshall T, Thangaratinam S, Thomas GN, Nirantharakumar K, Adderley NJ. Association between the reproductive health of young women and cardiovascular disease in later life: umbrella review. BMJ 2020;371:m3502. 33028606 10.1136/bmj.m3502 PMC7537472

[deac250-B30] Oksjoki R , Kovanen PT, Meri S, Pentikainen MO. Function and regulation of the complement system in cardiovascular diseases. Front Biosci 2007;12:4696–4708. 17485406 10.2741/2419

[deac250-B31] Olkowicz M , Czyzynska-Cichon I, Szupryczynska N, Kostogrys RB, Kochan Z, Debski J, Dadlez M, Chlopicki S, Smolenski RT. Multi-omic signatures of atherogenic dyslipidaemia: pre-clinical target identification and validation in humans. J Transl Med 2021;19:6. 33407555 10.1186/s12967-020-02663-8 PMC7789501

[deac250-B32] Perez-Riverol Y , Bai J, Bandla C, García-Seisdedos D, Hewapathirana S, Kamatchinathan S, Kundu DJ, Prakash A, Frericks-Zipper A, Eisenacher M et al The PRIDE database resources in 2022: a hub for mass spectrometry-based proteomics evidences. Nucleic Acids Res 2022;50:D543–D552. 34723319 10.1093/nar/gkab1038 PMC8728295

[deac250-B33] Piltonen T , Puurunen J, Hedberg P, Ruokonen A, Mutt SJ, Herzig KH, Nissinen A, Morin-Papunen L, Tapanainen JS. Oral, transdermal and vaginal combined contraceptives induce an increase in markers of chronic inflammation and impair insulin sensitivity in young healthy normal-weight women: a randomized study. Hum Reprod 2012;27:3046–3056. 22811306 10.1093/humrep/des225

[deac250-B34] Redinger RN. The coming of age of our understanding of the enterohepatic circulation of bile salts. Am J Surg 2003;185:168–172. 12559450 10.1016/s0002-9610(02)01212-6

[deac250-B35] Ricklin D , Hajishengallis G, Yang K, Lambris JD. Complement: a key system for immune surveillance and homeostasis. Nat Immunol 2010;11:785–797. 20720586 10.1038/ni.1923 PMC2924908

[deac250-B36] Silva JC , Denny R, Dorschel C, Gorenstein M V, Li G-Z, Richardson K, Wall D, Geromanos SJ. Simultaneous qualitative and quantitative analysis of the *Escherichia coli* proteome. Mol Cell Proteomics2006a;5:589–607. 16399765 10.1074/mcp.M500321-MCP200

[deac250-B37] Silva JC , Gorenstein MV, Li GZ, Vissers JPC, Geromanos SJ. Absolute quantification of proteins by LCMSE: a virtue of parallel MS acquisition. Mol Cell Proteomics2006b;5:144–156. 16219938 10.1074/mcp.M500230-MCP200

[deac250-B38] Šimják P , Cinkajzlová A, Anderlová K, Kloučková J, Kratochvílová H, Lacinová Z, Kaválková P, Krejčí H, Mráz M, Pařízek A et al Changes in plasma concentrations and mRNA expression of hepatokines fetuin A, fetuin B and FGF21 in physiological pregnancy and gestational diabetes mellitus. Physiol Res 2018;67:S531–S542. 30484680 10.33549/physiolres.934017

[deac250-B39] Sitruk-Ware R , Nath A. Characteristics and metabolic effects of estrogen and progestins contained in oral contraceptive pills. Best Pract Res Clin Endocrinol Metab 2013;27:13–24. 23384742 10.1016/j.beem.2012.09.004

[deac250-B40] Sitruk-Ware R , Plu-Bureau G, Menard J, Conard J, Kumar S, Thalabard JC, Tokay B, Bouchard P. Effects of oral and transvaginal ethinyl estradiol on hemostatic factors and hepatic proteins in a randomized, crossover study. J Clin Endocrinol Metab2007a;92:2074–2079. 17374706 10.1210/jc.2007-0026

[deac250-B41] Sitruk-Ware R. Hormonal contraception and thrombosis. Fertil Steril 2016;106:1289–1294. 27678035 10.1016/j.fertnstert.2016.08.039

[deac250-B42] Sitruk-Ware RL , Menard J, Rad M, Burggraaf J, de Kam M, Tokay BA, Sivin I, Kluft C. Comparison of the impact of vaginal and oral administration of combined hormonal contraceptives on hepatic proteins sensitive to estrogen. Contraception2007b;75:430–437. 17519148 10.1016/j.contraception.2007.01.027

[deac250-B43] Stanczyk FZ , Archer DF, Bhavnani BR. Ethinyl estradiol and 17β-estradiol in combined oral contraceptives: pharmacokinetics, pharmacodynamics and risk assessment. Contraception 2013;87:706–727. 23375353 10.1016/j.contraception.2012.12.011

[deac250-B44] Tedder RS , Nelson M, Eisen V. Effects on serum complement of normal and pre-eclamptic pregnancy and of oral contraceptives. Br J Exp Pathol 1975;56:389–395. 1212421 PMC2072786

[deac250-B45] van Rooijen M , Hansson LO, Frostegård J, Silveira A, Hamsten A, Bremme K. Treatment with combined oral contraceptives induces a rise in serum C-reactive protein in the absence of a general inflammatory response. J Thromb Haemost 2006;4:77–82. 16409455 10.1111/j.1538-7836.2005.01690.x

[deac250-B46] Virtanen P , Gommers R, Oliphant TE, Haberland M, Reddy T, Cournapeau D, Burovski E, Peterson P, Weckesser W, Bright J et al; SciPy 1.0 Contributors. SciPy 1.0: fundamental algorithms for scientific computing in Python. Nat Methods 2020;17:261–272. 32015543 10.1038/s41592-019-0686-2 PMC7056644

[deac250-B47] Wang Q , Wurtz P, Auro K, Morin-Papunen L, Kangas AJ, Soininen P, Tiainen M, Tynkkynen T, Joensuu A, Havulinna AS et al Effects of hormonal contraception on systemic metabolism: cross-sectional and longitudinal evidence. Int J Epidemiol 2016;45:1445–1457. 27538888 10.1093/ije/dyw147 PMC5100613

[deac250-B48] Wang Y-D , Chen W-D, Moore DD, Huang W. FXR: a metabolic regulator and cell protector. Cell Res 2008;18:1087–1095. 18825165 10.1038/cr.2008.289

[deac250-B49] Westhoff CL , Petrie KA, Cremers S. Using changes in binding globulins to assess oral contraceptive compliance. Contraception 2013;87:176–181. 22795088 10.1016/j.contraception.2012.06.003 PMC3494777

[deac250-B50] Wiegratz I , Kutschera E, Lee JH, Moore C, Mellinger U, Winkler UH, Kuhl H. Effect of four different oral contraceptives on various sex hormones and serum-binding globulins. Contraception 2003;67:25–32. 12521654 10.1016/s0010-7824(02)00436-5

